# Epidemiology and biology of early onset colorectal cancer

**DOI:** 10.17179/excli2021-4456

**Published:** 2022-01-07

**Authors:** Anand Venugopal, John M. Carethers

**Affiliations:** 1 Division of Gastroenterology and Hepatology, Department of Internal Medicine, University of Michigan, Ann Arbor, Michigan, USA; 2 Department of Human Genetics and Rogel Cancer Center, University of Michigan, Ann Arbor, Michigan, USA

**Keywords:** colon cancer, rectal cancer, young onset, early onset, cancer genetics, diabetes mellitus, obesity, cancer risk factors, cancer treatment, cancer prevention, tumor microenvironment, inflammation, colonoscopy, cancer screening, genetic testing

## Abstract

Colorectal cancer (CRC) is the third leading cause of cancer-related mortality in men or women in the United States. Average-risk screening that begins at age 50 years has reduced incidence and mortality of CRC in those over 50 years of age, whereas CRC incidence in those under age 50 years (early onset colorectal cancer (eoCRC)) has recently and dramatically increased. In this review, we summarize the recent literature including risk factors for eoCRC, differences in clinicopathologic presentation and outcomes in eoCRC, and emerging evidence regarding the molecular pathways that are altered in eoCRC compared to later onset CRC (loCRC). Epidemiologic studies of eoCRC show predominance in distal colon and rectum, and association with several modifiable risk factors, including diabetes, obesity, diet, sedentary time, alcohol consumption and smoking. Data regarding potential risk factors of prior antibiotic exposure and microbiome alterations or direct carcinogen exposure are still emerging. Aggressive clinicopathologic features of eoCRC at presentation may be due to delay in diagnosis or more aggressive tumor biology. EoCRC outcomes are similar to loCRC when matched for stage, but overall mortality is greater due to higher frequency of advanced disease at a younger presentation, with more life-years lost. There are only few molecular evaluations of eoCRC to date, with findings of potential increase in *TP53* and *CTNNB1* somatic mutation and decrease in *APC*, *KRAS* and *BRAF* somatic mutation, compared to loCRC. Other findings include LINE-1 hypomethylation, absence of microsatellite instability (MSI-H), presence of chromosomal instability (CIN) or microsatellite and chromosomal stability (MACS). These studies are only now emerging and have not yet identified a specific molecular signature defining eoCRC. Further research evaluating genetic and molecular differences as well as environmental triggers for eoCRCs should provide a clearer understanding to inform targeted screening for pre-symptomatic at-risk younger individuals.

## Abbreviations

ACG American College of Gastroenterology

ACS American Cancer Society

AGA American Gastroenterological Association

AHEI Alternative Healthy Eating Index

AMED alternative Mediterranean diet

APC adenomatous polyposis coli

BMI body mass index

CIMP CpG Island Methylator Phenotype

CIN chromosomal instability

CRC colorectal cancer

DASH dietary approaches to stop hypertension

eoCRC early onset CRC

LINE-1 long interspersed nuclear element-1

loCRC later onset CRC

MACS microsatellite and chromosome stability

MMR DNA mismatch repair

MSI-H microsatellite instability-high

MSS microsatellite stable

NSAID non-steroidal anti-inflammatory drug

PCR polymerase chain reaction

SEER Surveillance, Epidemiology, and End Results

USPSTF US Preventative Services Task Force 

## Introduction

Colorectal cancer (CRC) is the third leading cause of cancer-related mortality in the United States in either men or women (Siegel et al., 2021[101]). Population-wide, average-risk CRC screening was initiated in the mid-1990s for all adults aged 50 years or older. Subsequently, CRC incidence and mortality for later onset CRCs (loCRCs) in those over age 50 years have progressively decreased over the last two decades (Siegel et al., 2020[102]). On the other hand, multiple studies have revealed an alarming phenomenon where the incidence of CRC in those younger than age 50 years, have been progressively increasing (Siegel et al., 2021[101], 2020[102], 2017[103]; Cavestro et al., 2018[29]; Bhandari et al., 2017[13]; Carethers, 2016[26]). 

As the phenomenon of an increasing incidence of early-onset CRC (eoCRC) has become more apparent, so too have the number of investigations attempting to further characterize the increase. Multiple epidemiological studies have demonstrated the progressive increase in eoCRC incidence as well as correlations between modifiable risk factors and eoCRC, but the underlying etiology of the rising incidence of eoCRC has yet to be determined. Relatively few studies have been published informing any molecular or somatic genetic differences in eoCRC; examining these differences allows for viewing the phenomenon of eoCRC through a different lens. Identifying potential molecular pathways that drive eoCRC formation or propagation can not only shed new light on pathogenic mechanisms, but also may reveal an etiologic cause of eoCRC and inform risk stratification to target at-risk patients to mitigate the occurrence of eoCRC. This review focuses on the most recent literature regarding the epidemiology, risk factors and molecular biology of eoCRC, with particular focus on what is known about how identified molecular and genetic changes of eoCRC differ compared to late onset CRC (loCRC).

## Epidemiology and Definitions of eoCRC

**Figure 1:**
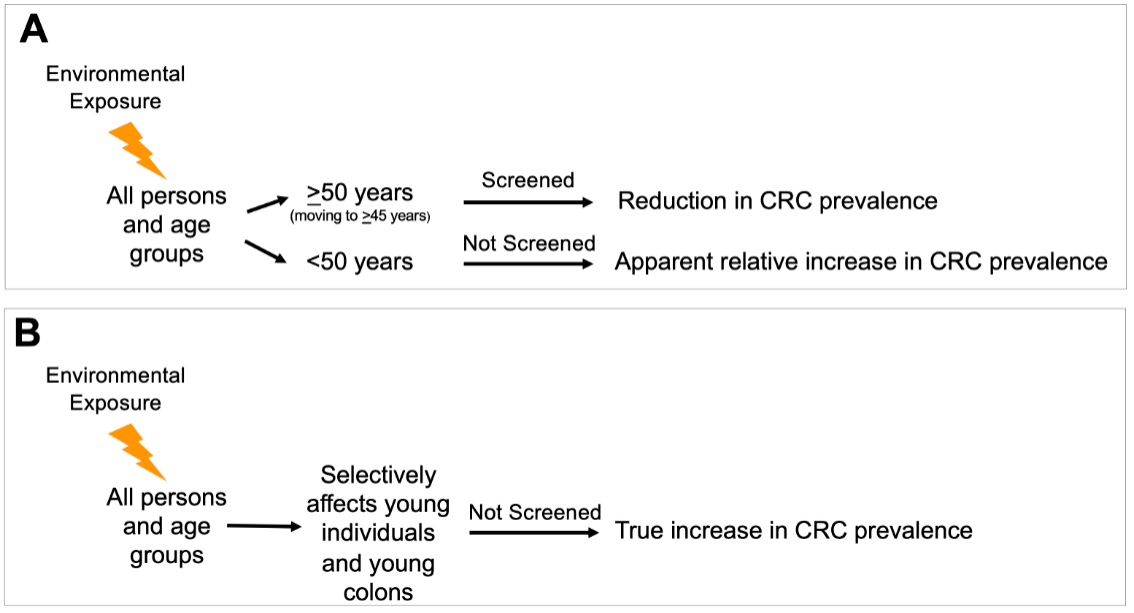
Possible epidemiological scenarios accounting for the observed increased incidence of early onset colorectal cancer. (A) An environmental exposure occurs in the total population, but an increase in colorectal cancer is not seen in patients that are screened, with an apparent increase in patients solely because that group is not screened. This scenario might imply genetic and epigenetic factors and pathways are like those observed in later onset colorectal cancer. (B) An environmental exposure occurs, but the exposure selectively affects younger populations over older populations (through accelerated pathogenesis), providing a true increase in colorectal cancer in that population without affecting older populations. This pathway might imply genetic and epigenetic factors and pathways that are distinct from those observed in later onset colorectal cancer. This scenario also implies that tumor initiation and possibly tumor progression are accelerated over the typical progression timeframes observed for later onset colorectal cancer.

Since implementation of average-risk screening for average risk adults age 50 years or older in the mid-1990s, the incidence and mortality of CRC overall has been steadily decreasing (Siegel et al., 2021[101], 2020[102]). The sole criterium for commencing screening of average-risk individuals was the non-modifiable risk factor of age (Winawer et al., 1997; Carethers, 2018[21]). Since then, the incidence of CRC as well as its associated mortality, both in those above 50 years of age and overall have continued to decrease, with most recent trends showing the average annual percentage change (AAPC) in incidence of CRC in those between 50 and 65 years of -0.7 from 2007 to 2016, and -4.0 in those age 65 or over (Siegel et al., 2020[102]). This is in contrast to the rise in eoCRCs. There could be one of two possibilities for this observation (Figure 1(Fig. 1)). It is apparent from the epidemiology that the rise in eoCRC cases is environmentally triggered and not genetic evolution. The apparent rise could affect all ages within the population, but an increase in CRC is not seen in older patients that are screened due to the preventative effects of screening colonoscopy, leading to an apparent increase in eoCRC patients solely because that group is not screened. Alternatively, an environmental exposure selectively affects younger populations over older populations, providing a true increase in eoCRC patients without affecting older populations.

### Rationale for <50 years of age as the age threshold in defining eoCRC

From 1975 to 1990, the incidence of eoCRC was trending downward (Siegel et al., 2017[103]; Murphy et al., 2018[77]), but over the past three decades, there has been a consistent, steady increase in both the incidence and mortality from CRC for the population below the age threshold for average-risk screening (Siegel et al., 2021[101], 2020[102], 2017[103]; Cavestro et al., 2018[29]; Bhandari et al., 2017[13]; Carethers, 2016[26]). At present, patients with eoCRC make up approximately 1 in 8 new CRC diagnoses. The largest burden of eoCRC is among individuals age 40-49 years, accounting for 75 % of the eoCRC incidence (Murphy et al., 2017[76]; You et al., 2012[120]), with a “catch-up” effect in those age 50-52 years at the time of screening initiation because a portion of CRC in the age 50-52-year group could have been detected earlier with onset of earlier screening (Siegel et al., 2020[102]). For this reason, the vast majority of eoCRC studies utilize an age cut-off of 50 years as this is the age where the majority of adults initiate CRC screening, and is also the inflection point for the age-dependent change in CRC incidence (likely occurs around age 51-52) (Siegel et al., 2020[102]; Winawer et al., 1997[117]). For the purposes of this review, eoCRC is defined here similar to most published studies, with an age threshold of 50 years (Table 1(Tab. 1)). For the past three decades for average-risk Americans, most professional societies recommend initiation of population-wide CRC screening for all adults at the age of 50 years. For the purposes of this review, later onset colorectal cancer (loCRC) is defined similarly to other literature with an age threshold of ≥50 years. 

### Known risk factors to lessen the screening age or accelerate screening frequency

High risk screening of patients with CRC is prompted by the presence of non-modifiable and well-established risk factors for CRC. Family history of CRC, family history of advanced adenoma, presence of a genetic or inherited cancer syndrome, personal history of adenoma and personal history of inflammatory bowel disease all prompt accelerated colonoscopy schedules for CRC screening (Shaukat et al., 2021[99]). Some studies have estimated that 25 % of current eoCRC cases could have been prevented through recognition of the family history as a risk factor and subsequent timely initiation of high-risk screening (Stanich et al., 2021[105]). Additional risk factors that have received conditional recommendations for initiation of early screening include African American race, history of abdominal or pelvic radiation for prior malignancy, or cystic fibrosis (Hadjiliadis et al., 2018[48]; Rex et al., 2009[90], 2017[89]; Gini et al., 2019[44]; Carethers, 2015[25]). While increased recognition and stricter adherence screening guidelines for high-risk individuals will likely reduce the incidence of eoCRC, it will not completely abrogate the rising incidence of eoCRC. Identification of additional risk factors that significantly contribute to eoCRC will be key in order to completely understand and mitigate the observed rise in incidence. 

The idea that the increasing incidence of eoCRC is caused by the increased utilization of colonoscopy is highly doubtful. Evidence shows that the eoCRC incidence continued to increase after the year 2008 when presumably, due to changes in healthcare practice patterns and economic recession, colonoscopy utilization decreased (Murphy et al., 2017[76]). Additionally, colonoscopy involves detection of adenomatous (pre-cancerous) polyps with subsequent polypectomy, and assuming a typical adenoma-to-adenocarcinoma transition sequence and dwell time, polypectomy would remove the pre-cancerous lesion prior to malignant transformation. Indeed, colonoscopy is one of the few truly “preventative” cancer screening measures employed that aids both the early detection of adenomas and cancers as well as prevents the onset of malignancy. In concordance with this, a recent study demonstrated that adenomas have been increasingly detected in the under 50 years of age population receiving colonoscopies for diagnostic reasons (Yip et al., 2021[119]). Lastly, multiple studies show that the diagnosis of eoCRC is delayed compared to loCRC due to under-recognition of alarm symptoms that normally triggers a diagnostic colonoscopy (Di Leo et al., 2021[39]; Taggarshe et al., 2013[109]; Scott et al., 2016[98]). 

### International trends for eoCRC

Much of the eoCRC trend data comes from the Surveillance, Epidemiology and End Results (SEER) registries, which accounts for patients within specific regions in the United States. Studies derived from international registries from other developed nations show similar trends in eoCRC as the U.S. (Lu et al., 2020[72]; Siegel et al., 2019[104]). In some areas of the world, eoCRC incidence trends have been hard to evaluate because of the lack of well-maintained registries. Interestingly, there are a few developed countries that demonstrate a decline in eoCRC. Specifically, Austria, Italy and Lithuania show persistent decreases in eoCRC rates. While it is not entirely clear why these countries demonstrate a declining eoCRC incidence as compared to other developed countries, Italy and Austria initiate CRC screening at age 44 and 40 respectively (Siegel et al., 2019[104]). 

### Updated screening guidelines to initiate at-risk CRC screening

Changes in eoCRC incidence has prompted evaluation of established CRC screening recommendations. Updated modeling studies using recent incidence rates of CRC suggest that average risk screening should be initiated at age 45 years because of higher than historical incidence rates of CRC among individuals aged 45-49 years (Peterse et al., 2018[87]; Meester et al., 2018[73]). These contemporary models prompted the American Cancer Society to issue a qualified recommendation for a new threshold to initiate CRC screening at the age of 45 years (Wolf, et al., 2018[118]). Later, the American Gastroenterological Association and the United States Preventative Services Task Force reiterated the same recommendations for earlier initiation of CRC screening (USPSTF et al., 2021[113]). The prospect of commencing CRC screening at age 45 years has generated some controversy, particularly around the focus of resource allocation to the 35 % of at-risk individuals above 50 years of age who are not currently screened as compared to the opening up of a greater pool of individuals that are aged 45-49 years. Ideally if we understood the biological reason for the epidemiological data showing this rise in eoCRC, we could more accurately risk stratify patients in the under-50 years age group to pre-symptomatically target screening resources aside from solely using age as the demarcating and deciding variable that determines the onset of at-risk population wide screening. Lastly, as more widespread adoption of age 45 years as the screening threshold occurs, this may prompt an alteration to the definition of eoCRC to that of age 45 or 40 years, or even lower.

The above recommendation (and controversy) to initiate screening at age 45 years for all average risk individuals due to the uptick in prevalence of eoCRC in the population is reminiscent of the same regarding longstanding epidemiological evidence for younger onset of CRC among African Americans, and the recommendations by some medical societies to commence earlier screening for African Americans (Kupfer et al., 2015[65]; Carethers, 2021[24]; Ashktorab et al., 2017[8]; Zavala et al., 2021[121]). Broad societal implementation for the total population for initiating screening at age 45 years might be an easier and uniform message to providers and patients but could constrain resources and could be more costly to society than an informed, targeted approach applied to higher risk individuals that are identified because of an understanding of the epidemiology and biology of the disease. Further, data is lacking on the optimal screening test(s) to be used for this expanded population pool that begins at age 45 years, as well as the added approach and costs to potential genetic testing among these patients who may be identified through screening in having advanced colorectal neoplasia (Carethers and Stoffel, 2015[28]; Stoffel and Carethers, 2020[106]; Carethers, 2020[22]).

## Unique Aspects of Clinical and Pathologic Presentation and Outcome for eoCRC Patients

Patients with eoCRC have differing clinical presentations, pathologic features and outcomes as compared to patients with loCRCs; these aspects are important considerations for both clinicians and scientists to fully evaluate the mystery of the eoCRC rise (Table 2(Tab. 2)).

Unlike patients with loCRCs where 65 % of the at-risk population is screened, the clinical presentation differs for most younger patients who, despite progression to presentation with alarm symptoms, are often initially ignored by providers due to their younger-than-50 years of age and that which may result in a delay of their diagnosis (Di Leo et al., 2021[39]; Taggarshe et al., 2013[109]; Scott et al., 2016[98]). Patients with eoCRCs are associated with later cancer stage at presentation; they present with more advanced stage III or stage IV disease (You et al., 2012[120]; O'Connell et al., 2004[82]; Saraste et al., 2020[96]; Kneuertz et al., 2015[63]; Burnett-Hartman et al., 2019[19]). It is unclear, however, to the degree in which the delay in diagnosis contributes to advanced stage disease at presentation versus aggressiveness of biological behavior. Patients with eoCRC are more often diagnosed after presenting with rectal bleeding, as eoCRC is most common in the rectosigmoid portion of the colon and differ from symptoms such as bowel obstruction in the case of more proximal colon tumors (Myers et al., 2013[79]; Riaz et al., 2017[91]). The overall time of delay in diagnosis is not definitively established, but it is likely a combination of patient- and provider-related factors. One study demonstrated that in patients presenting with rectal cancer, the time to initiate treatment from onset of symptoms was an average of 217 days in patients under 50 years of age, compared to 29.5 days for patients over 50 years of age (Scott et al., 2016[98]). Interestingly, the same study also revealed that the largest component to delay in diagnosis was from symptom onset to presentation to a primary care provider (approximately 6-fold longer compared to older CRC patients) and subsequent referral to specialist for diagnostic evaluation was a smaller component (approximately 1.5-fold longer) suggesting both patient and physician factors for under-recognition of symptoms for CRC (Scott et al., 2016[98]). 

Patient eoCRCs show aggressive histologic features such as signet-ring histology, poor differentiation, or perineural or venous invasive features compared to loCRCs or eoCRCs associated with an identified germline mutation (You et al., 2012[120]; Burnett-Hartman et al., 2019[19]; Chang et al., 2012[30]; Stoffel et al., 2018[107]). Chen et al. conducted a retrospective study at a single center showing that the cancer stage at diagnosis was inversely proportional to time to diagnosis, and when controlling for stage at diagnosis, there was no significant difference in workup duration (Chen et al., 2017[32]).

The one common feature that is consistent across multiple studies of sporadic (non-germline) eoCRCs is that they most frequently occur in distal colon and rectum, with any incidence of proximal disease associated with older age of onset (You et al., 2012[120]; Burnett-Hartman et al., 2019[19]; Siegel et al., 2009[100]; Willauer et al., 2019[116]). The frequency of rectal cancers had higher annual percentage change (+3.9 %) than colon cancers (+2.7 %) among young patients (You et al., 2012[120]). There may be heterogeneity in the pathogenesis of rectal versus colon cancers that predispose younger patients to develop distal disease (Carethers, 2011[23]). One report suggests that patients with eoCRC have likelihood of a second primary malignancy with highest association in the first 6-11 months following the CRC diagnosis. In comparison, patients with loCRCs show significantly lower rates for a second primary malignancy, implying potential risk factors or exposures contributing to the pathogenesis of eoCRCs may act through carcinogenic mechanisms in multiple tissues (Tiritilli and Ko, 2021[111]).

There are no current guidelines or evidence supporting any specific preventive, surveillance or treatment modality for patients with eoCRC over loCRC. Clinical patterns in patients with eoCRC do tend to differ as listed above, and by the nature of their younger presentation and potential lifespan remaining, eoCRC patients are more likely receive aggressive surgical resections (such as metastatic resections), multimodal chemotherapy and immunotherapy, than their older counterparts (Kneuertz et al., 2015[63]; Burnett-Hartman et al., 2019[19]; Cheng et al., 2021[34]). Most studies note that when matched for stage and other clinical presenting factors, eoCRC patients have modestly better prognosis than later onset patients, although it is unclear the degree to which additional comorbidities of loCRC patients play into this (Burnett-Hartman et al., 2019[19]; Cheng et al., 2021[34]; Perrott et al., 2020[86]; Chen et al., 2020[33]; Georgiou et al., 2019[43]). One survey of U.S.-based patients with eoCRCs demonstrated that they were more likely to have advanced disease, and irrespective of staging, younger patients were more apt to receive systemic chemotherapy (Kneuertz et al., 2015[63]). Despite this, there is no demonstrated increase in patient survival of eoCRC patients over loCRC patients. This lack of improved survival may be attributed to either overtreatment coupled with lack of response, or the presence of a more aggressive underlying tumor biology with overtreatment required to potentially elicit an improved survival response (Kneuertz et al., 2015[63]). One multi-center evaluation shows that younger patients are more likely to have extensive lymph node examination and dissection and receive systemic chemotherapy or immunotherapy due to advanced stage and aggressive histology at presentation, and when adjusted for these factors, eoCRC patients had reduced CRC-specific death (Burnett-Hartman et al., 2019[19]). On the other hand, a UK-based retrospective single center study demonstrated that among eoCRC patients with stage IV disease, there is an inverse relationship between age of onset and median overall survival, implying that earlier onset disease is driven by a more rapidly progressive or aggressive tumor biology (Georgiou et al., 2019[43]). A large U.S. registry-based study showed that patients with eoCRC possessed higher rates of overall mortality, but when adjusted for stage, eoCRC patients had slightly better overall survival (Cheng et al., 2021[34]). Importantly, the survival benefit among patients with eoCRC was most pronounced with stage I and stage II disease, implying that early diagnosis in eoCRC may have a significant impact on improving overall mortality from eoCRC (Cheng et al., 2021[34]). 

These studies highlight significant knowledge gaps in our understanding of eoCRC. First, it is not clear whether more aggressive histologic features and higher frequency of advanced disease at presentation can be entirely explained by delays in diagnosis, or if there is an accelerated pathogenesis among younger populations that propagate a more rapid progression. Second, eoCRC patients typically receive more aggressive treatment regimens, and it is unclear if lack of significant improvements in survival are due to lack of efficacy of these more aggressive treatment regimens, or if these treatment regimens are necessary to overcome more aggressive tumor biology to match outcomes of loCRC patients. Lastly, there is very limited data regarding survivorship and surveillance following treatment, considering that risk factors that contributed to the accelerated pathogenesis may warrant more intensive and longer post-treatment surveillance duration. These questions highlight a significant knowledge gap in eoCRC regarding the underlying pathogenic steps and differences in cancer biology for eoCRC.

One important point is that while patient mortality from loCRC is trending downward with an annual percentage change of -1.8 %, the patient mortality from eoCRC is increasing by an annual percentage change of +1.3 % (Siegel et al., 2020[102]). The change in mortality appears to be driven by increased frequency of advanced disease. Although patients with eoCRC may have modestly improved survival outcomes when matched for stage of patients with loCRC, this is overshadowed by the increased frequency of late-stage cancers causing both increased overall mortality and greater life-years lost due to the early age of diagnosis. Considering this and the fact that a survival benefit in eoCRC appears most prominent with early-stage disease, the argument for early detection becomes stronger and paramount.

## Modifiable Risk Factors Associated with eoCRC

Modern next generation sequencing indicates that eoCRC is a sporadic disease after eliminating the 20 % of younger-than-50 years of age that possesses a germline mutation defining a specific disease, with the remaining 80 % (eoCRC) having no revealing genetic mutation (Stoffel et al., 2018[107]). The increase in incidence of eoCRC corresponds with the birth cohort of individuals born after the year 1960 and beyond, sharing progressively higher risk of eoCRC compared to older generations (Stoffel and Murphy, 2020[108]). While the mechanism behind the increasing incidence is still not clearly established, there are several known modifiable risk factors that are implicated for the pathogenesis of eoCRC (Table 2(Tab. 2)).

### Obesity or high Body Mass Index

There is an expanding body of evidence that implicates obesity, low physical activity, Western dietary patterns and sedentary lifestyle all as factors associated with risk for eoCRC. Using data from the Nurses' Health Study II, obesity (measured by body mass index (BMI) > 30) in early adulthood was associated with a nearly two-fold increase in the relative risk for eoCRC (Liu et al., 2019[70]). Importantly, there was proportionate increase in the relative risk with increasing BMI as well as with weight gain since early adulthood (Liu et al., 2019[70]). Meta-analyses demonstrate similar findings, with a proportional increase in eoCRC risk associated with increasing BMI (Hidayat et al., 2018[50]; Li et al., 2021[67]; O'Sullivan et al., 2021[85]). Similarly, the rise of eoCRC corresponds to increasing surgical resections in obese patients (Hussan et al., 2020[55]). Paradoxically, a case-control study of US veterans found that being overweight or having an obese-range BMI conferred decreased risk for eoCRC, while being underweight increased risk (Low et al., 2020[71]). This finding was primarily attributed to using BMI close to the time of colonoscopy rather than the BMI during early adulthood. In line with this consideration, weight loss >5 kg over a 5-year period prior to time of colonoscopy was positively associated with eoCRC. This finding also highlights the potential pitfall of analyzing risk factors close to the time of diagnosis rather than in early adulthood when potential initiating steps as a result of that risk factor might occur.

### Type II Diabetes Mellitus

Closely associated with obesity, type II diabetes mellitus has been shown to be a risk factor for eoCRC (Stoffel and Murphy, 2020[108]; de Kort et al., 2017[37]; Ali Khan et al., 2020[4]; Mikaeel et al., 2021[74]). A meta-analysis demonstrated that independent of age, diabetes is associated with increased risk for CRC, with a relative risk of 1.3 (Larsson et al., 2005[66]). The increase in eoCRC mirrors global trends in the prevalence of diabetes mellitus, with 30 million people with diabetes mellitus estimated in 1964 as compared to 171 million people with diabetes 40 years later (Ogurtsova et al., 2017[83]). With growth of the global elevation of childhood obesity, the worldwide prevalence of diabetes is anticipated to continue to increase over the next few decades (Dabelea et al., 2014[36]). Metformin, a medication commonly used for the treatment and prevention of diabetes, has been shown to be a chemoprevention agent for adenoma formation (Hosono et al., 2010[53]; Higurashi et al., 2016[51]). A multicenter, randomized, double-blinded trial for patients who had adenomas who underwent treatment with metformin or placebo for 1 year showed decreased adenoma formation on repeat endoscopy (Higurashi et al., 2016[51]). 

A Swedish registry study revealed that the cumulative risk of CRC was similar in patients with diabetes as compared to those with a family history of CRC without diabetes (Ali Khan et al., 2020[3]). Patients with diabetes were observed to have an accelerated cumulative risk for CRC by approximately 5 years earlier as compared to the average population (Ali Khan et al., 2020[3]). A similar Swedish cohort study observed that a diabetes diagnosis before the age of 50 years provided a 1.9-fold increased risk of developing eoCRC, with the lifetime risk of CRC with diabetes nearly matched to that of patients who had a family history of CRC (Ali Khan et al., 2020[4]). These findings are notable, as current screening guidelines advocate for high-risk screening to be initiated at age 40 years based on family history as the risk factor; diabetes may confer an equivalent risk that is not yet considered in guidelines that inform the medical decision of when to initiate screening.

### Diet

Highly intertwined with obesity and diabetes, lifelong high fat and high caloric diets provide increased risk for CRC. Most of the evidence centers on dietary patterns that include high consumption of red meats, processed meats and sugary beverage intake with higher risk for CRC, whereas higher consumption of fruits, vegetables, dietary fiber, calcium and yogurt are associated with decreased CRC risk (O'Sullivan et al., 2021[85]; Chang et al., 2021[31]; Hur et al., 2021[54]; Nguyen et al., 2021[80]; Zheng et al., 2021[122]; Veettil et al., 2021[114]). The quality of evidence is low in many of these retrospective studies and is additionally difficult to interpret due to the self-reporting nature of diets at or near the time of diagnosis. As pro-carcinogenic contributions from diet and its metabolites likely begins to occur decades prior to the actual clinical presentation of CRC, self-reported diets at or near the time of diagnosis are difficult to interpret regarding their direct contribution towards CRC risk. 

The Nurses' Health Study II used regular intervals of standardized dietary questionnaires over a 20-year period, providing longitudinal evaluation of dietary impact on eoCRC risk. From this data, Westernized diets are associated with increased risk of early-onset adenomas, whereas longitudinal data from the Dietary Approaches to Stop Hypertension (DASH), Alternative Mediterranean (AMED), and Alternative Healthy Eating Index-2010 (AHEI-2010) studies show that their associated diets were all protective for early-onset adenomas (Zheng et al., 2021[122]). These observations were most related to distal disease, which taken together with the higher prevalence of distal location in eoCRC, might demonstrate a relatively strong association between dietary patterns and eoCRC. 

Using the Nurses' Health Study II dataset, Nguyen et al. reported that a sulfur microbial diet (diet with high intake of processed meats, defined by increased abundance of microbial species associated with sulfur metabolism), was associated with increased risk of advanced adenoma formation in those under age 50 years (Nguyen et al., 2021[80]). Also from the Nurses' Health Study II, the consumption of >2 servings per day of sugar-sweetened beverages was associated with a RR of 2.18 for eoCRC as compared to those who consumed <1 serving per week (Hur et al., 2021[54]). There was a statistically significant incremental ingestion trend for elevated risk, and replacement with artificially sweetened beverages was associated with a decreased risk of eoCRC (Hur et al., 2021[54]). It is important to note that these studies are limited as they come from analysis of a single data set of women and uses adenomas as a surrogate for CRC formation. Chang et al. instead used self-reported data recalled from 2 years prior to diagnosis, and reported Western diets having a similar risk profile for development of eoCRC (Chang et al., 2021[31]). 

### Physical activity

Physical activity and the underlying mechanisms by which it modulates carcinogenesis are highly connected with adiposity, diet, and diabetes, and some studies demonstrate that sedentary lifestyle as compared to active physical activity is associated with elevated risk for eoCRC (O'Sullivan et al., 2021[85]; Chang et al., 2021[31]). When adjusted for obesity, diabetes, smoking, alcohol intake, dietary habits, physical activity and BMI, sedentary behavior (characterized by TV viewing time >14 hours per week) conferred a relative risk of 1.7 as compared to <7 hours of TV viewing per week (Nguyen et al., 2018[81]). This suggests that sedentary time alone serves as a risk factor for colorectal carcinogenesis in the young, independent of obesity, diet, diabetes, and physical activity.

### Alcohol and tobacco use

Additional lifestyle exposures that have been associated with CRC development include alcohol and tobacco use. Alcohol-related liver disease has been progressively increasing, particularly in younger adults (Tapper and Parikh, 2018[110]). A meta-analysis showed that alcohol consumption provides a dose-related increased risk for CRC, with a relative risk of 1.52 for heavy alcohol consumption (Fedirko et al., 2011[41]). Similar findings have been observed in a meta-analysis regarding eoCRC, showing a relative risk of 1.71 with heavy alcohol usage (O'Sullivan et al., 2021[85]). Smoking is a risk factor for the development of CRC and contributes to advanced adenoma formation in the young (O'Sullivan et al., 2021[85]; Kim et al., 2016[62]; Botteri et al., 2008[15][16]; Liang et al., 2009[68]). Botteri et al. showed through meta-analysis that cigarette smoking, in a dose-dependent fashion, caused a modest relative risk of 1.18 for current and past smokers (Botteri et al., 2008[16]). Current smokers had the highest relative risk of 2.14 for adenomas, and the relative risk became stronger for advanced adenoma formation (Botteri et al., 2008[16]). O'Sullivan et al. noted through meta-analysis a trend towards increased risk from smoking for eoCRC, but while the risk did not reach statistical significance, there was significant heterogeneity between studies used in the meta-analysis (O'Sullivan et al., 2021[85]). 

A retrospective Korean cohort analysis demonstrated that eoCRCs as well as advanced colorectal neoplasms (defined by adenomas > 10 mm, villous histology, high grade dysplasia or adenocarcinoma) in those under age 50 years was associated with diabetes, obesity, and smoking (Kim et al., 2016[62]). This finding suggests that modifiable risk factors may play a role in early pathogenesis for eoCRC, perhaps at the stage of adenoma formation. The similar findings of risk factors from this Korean cohort aligns with findings from U.S. and European cohorts and implies that these risk factors are universally applicable across populations.

### Aspirin and NSAID use

Long-term use of non-steroidal anti-inflammatory drugs (NSAIDs) and aspirin use in prospective trials have been shown to be protective for adenoma and CRC formation, and current American College of Gastroenterology (ACG) guidelines provide a conditional recommendation for aspirin use in those 50-64 years old for CRC prevention (Shaukat et al., 2021[99]; Chubak et al., 2016[35]; Rothwell et al., 2010[93]; Koi et al., 2020[64]). While significant evidence is lacking for modulation of eoCRC risk, and no prospective data exists currently, observational data from U.S. veterans showed that aspirin use is protective for eoCRC (Low et al., 2020[71]). The patient population in this study could limit the generalizability of this finding, which consisted of predominantly male veterans with high rates of obesity.

## Proposed Mechanisms for the Pathogenesis of eoCRC

### Hyperglycemia, hyperinsulinemia and insulin resistance

There appears to be considerable interplay between mechanisms associated with colorectal pathogenesis and risk factors such as obesity, diabetes and physical inactivity. While no studies yet directly link these risk factors with any pathogenic pathways that might specifically drive eoCRC, there are proposed mechanisms that help explain the associations between diabetes and obesity and eoCRC. The downstream effects of diabetes and obesity such as hyperglycemia, hyperinsulinemia, insulin resistance and increased adipocytokines generate carcinogenic effects through alterations in cellular signaling and energy metabolism. Hyperinsulinemia and hyperglycemia have been shown to promote intestinal cell proliferation through the Wnt/β-catenin signaling pathway and through stimulation of MAPK pathways through insulin-like growth factor receptor (Grega et al., 2021[46]). Hyperglycemia can generate oxidative stress and alter cellular energy metabolism to promote cancer cell proliferation, as well as have direct inflammatory contributions from advanced glycation end-products and increased reactive oxygen species (Grega et al., 2021[46]). Abdominal obesity represents increased adipocyte tissue, which in turn serves as an endocrine reservoir generating adipokines such as leptin, adiponectin, TNF-α, IL-6, and circulating estrogens, all of which have been implicated in carcinogenesis (Avgerinos et al., 2019[10]; Murphy et al., 2018[78]; Grivennikov and Karin, 2011[47]; Tseng-Rogenski et al., 2020[112]). However, the overall effect of adiponectin and leptin are not clear, with a meta-analysis showing no significant risk from leptin for CRC, and the risk associated with adiponectin varies based on patient weight and gender (Wang et al., 2021[115]).

### Chronic inflammation

Localized inflammation is well-associated with CRC risk. Inflammatory bowel disease (IBD) is a known significant risk factor for the development of dysplasia and colorectal neoplasia (Jess et al., 2012[57]; Rubin et al., 2013[94]; Munakata et al., 2019[75]), prompting the strong and now standard recommendation to initiate high risk colorectal cancer surveillance in patients with longstanding ulcerative colitis (Kaltenbach et al., 2017[59]). Similarly, a state of subclinical chronic inflammation from increased concentration of circulating adipokines could generate an inflammatory environment that is permissive for eoCRC carcinogenesis through production of free radical species and upregulation of anti-apoptosis and proliferation pathways (Murphy et al., 2018[78]).

### Intestinal dysbiosis

Obesity, specific diets, and chronic inflammation can generate intestinal dysbiosis. One potential mechanism by which eoCRC may arise is through alterations in the gut microbiome. Specific intestinal bacterial species such as *Bacteroides fragilis*, *Fusobacterium nucleatum*, *Streptococcus bovis*, and *Escherichia coli*, can facilitate colorectal carcinogenesis through altering colonic integrity affecting systemic inflammatory responses, through toxin production, and through metabolic products of short chain fatty acids (SCFAs) and alterations in bile acid composition (Abdullah et al., 2021[1]; Okita et al., 2020; Dejea et al., 2018[38]). Hydrogen sulfide producing bacteria are associated with CRC; hydrogen sulfide has been shown to induce DNA damage and promote proliferation in human colon cancer cell lines (Attene-Ramos et al., 2007[9]; Scanlan et al., 2009[97]; Cai et al., 2010[20]), and diets that facilitate sulfide-producing microbes have been implicated in eoCRC (Nguyen et al., 2021[80]). Intestinal dysbiosis as well as alcohol intake can result in elevated acetaldehyde levels which have been implicated in direct DNA damage and telomere shortening *in vitro *(Harpaz et al., 2018[49]). While not specifically investigated in eoCRC, increased antibiotic use has been shown in meta-analyses to be weakly associated with increased CRC risk (Sanyaolu et al., 2020[95]; Aneke-Nash et al., 2021[5]) presumably through alterations in the gut microbiome. However, animal studies are conflicting regarding antibiotic use, showing both anti- and pro-carcinogenic effects through either depletion of natural gut flora leading to cancer progression, or depletion of cancer promoting microbes inhibiting cancer growth (Bullman et al., 2017[18]; Kaur et al., 2018[60]).

### Direct carcinogen exposure

In addition to alterations in the colonic immune microenvironment, gut microbiome, and cellular proliferation and energy metabolic pathways, some factors may contribute to the development of eoCRC through direct carcinogen exposure. For example, alcohol can be metabolized into acetaldehyde which then generates direct genotoxic effects on the colonic epithelium thus contributing directly to colorectal carcinogenesis (Johnson et al., 2021[58]). Similarly, red as well as processed meats (both typically enriched in Western diets) contain *N*-nitroso compounds, heterocyclic amines, and heme iron that can generate direct carcinogenic effects in colon epithelia (Veettil et al., 2021[114]; Bouvard et al., 2015[17]). Nicotine, a component of tobacco and electronic cigarettes, can alter cellular processes such as proliferation, migration, angiogenesis and apoptosis; cigarette smoke also results in systemic exposure of carcinogens through the systemic circulation and through the aerodigestive tract (Jensen et al., 2012[56]). 

## Reported Molecular Differences in eoCRC

Colorectal adenocarcinomas can be thought of as a heterogenous group of cancers that typically progressed through a defined set of molecular pathway alterations; these molecular pathways are well accepted and reviewed thoroughly elsewhere (Fearon, 2011[40]; Rodriguez-Salas et al., 2017[92]; Carethers and Jung, 2015[27]; Grady and Carethers, 2008[45]; Raeker and Carethers, 2020[88]). About 85 % of CRCs follow the chromosomal instability (CIN) pathway associated with initiating loss-of-function mutations in the adenomatous polyposis coli (*APC*) gene involved in the WNT/β-catenin signaling pathway, triggering histologic aberrant crypt foci formation. Subsequent progressive accumulation of mutations in *KRAS* and *TP53* transforms adenoma tissue into adenocarcinoma. About 15 % of sporadic CRCs possess mismatch repair (MMR) deficiency through suppression of *MLH1* expression by methylation of its promoter. MMR deficiency is observed by the lack of MLH1 protein expression with immunohistochemistry of CRC tissue, or via PCR of mono- and di-nucleotide microsatellites from cancer DNA to demonstrate the biomarker of microsatellite instability-high (MSI-H). MMR-deficient tumors possess hypermutated genomes, and with frameshift mutation of coding microsatellites, create high levels of neoantigens that triggers a protective immune response simultaneous with high expression of programmed death ligand or receptor. Patients with MMR-deficient tumors tend to have improved survival over patients with MMR-proficient tumors (matched for stage), and patients have additional survival benefit from immune checkpoint inhibitors. The CpG island methylator phenotype (CIMP) pathway overlaps with CIN or MSI-H pathways and accumulates methylation in the cancer genome; it is most associated with the pathogenesis of sessile serrated adenomas and adenocarcinomas.

Studies directly investigating alterations in the molecular profile of eoCRCs are limited. After the elimination of single Mendelian inherited germline mutations (~20 % of CRC patients under the age of 50 years (Stoffel et al., 2018[107])), the remaining 80 % of patients defined as eoCRC show some evidence of genetic risk association via polygenic risk scores (Archambault et al., 2020[7]). Archambault et al. determined the polygenic risk scores (utilizing 95 CRC-associated genes) and revealed that higher risk scores are inversely associated with age, implying that multiple low-penetrance polymorphisms might contribute genetic risk for eoCRC as compared to loCRC (Archambault et al., 2020[7]). 

**Figure 2:**
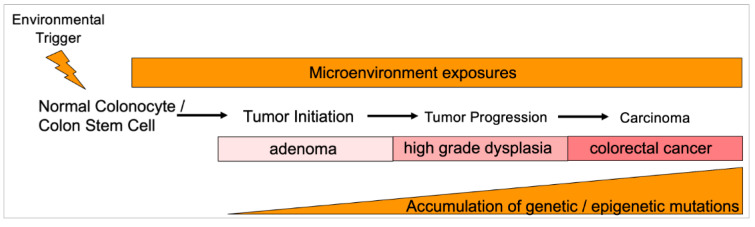
Hypothesized sporadic normal-to-adenoma-to-carcinoma progression in the colon for early onset colorectal cancer. A presumed environmental trigger in the microenvironment milieu helps create the conditions that trigger early existing and/or initiating genetic and epigenetic alterations that begin to propel a normal colonocyte or colon stem cell towards excessive proliferation. After neoplastic initiation, the local microenvironment intermittently or constantly continues to provide the conditions to propel further genetic and epigenetic alterations that are manifested by histologic changes, ultimately accumulating sufficient genomic changes to define it as a cancer and acquiring the capability of metastatic spread. The local microenvironment is highly influenced by diet and diet supplements, tobacco and alcohol use, aspirin and NSAID use, and metabolic pathways important in obesity regulation. Within the local microenvironment are multiple factors that may indirectly or directly influence epithelial behavior, including the makeup of the gut microbiome and its metabolites and toxins, levels and types of inflammatory cells, presence of oxygen and free radicals, and other potential carcinogens.

The accumulation of somatic mutations within eoCRCs may be altered compared to loCRCs (Figure 2(Fig. 2)). Kim et al. demonstrated with integration of multiple whole exome sequencing datasets that the prevalence of *TP53* mutations was higher in non-hypermutated eoCRC compared to loCRC, while the mutational frequency of *APC* and *KRAS* were significantly lower in eoCRC (Kim et al., 2021[61]). Similarly, among patients under age 40 years as compared to those over 60 years of age, somatic *TP53* and *CTNNB1* mutations occurred more frequently in the younger patients, and *APC*, *KRAS* and *BRAF* mutations occurred less frequently (Lieu et al., 2019[69]). On the contrary, using cell-free DNA sequencing from a large cohort of 1296 eoCRC and 4577 loCRC patients, there was increased frequency of sequence alterations in *TP53*, *APC*, *KRAS* and *PIK3CA* among eoCRC patients (Barzi et al., 2021[12]). The above studies evaluated mutational profiles in many of the commonly mutated, canonical pathways associated with CRC, but additional studies show that eoCRC may not follow any previously established pathways or may possess unique mutational aspects. Aitchison et al. showed that in a cohort of 25 eoCRC patients from New Zealand that *APC *mutation frequency was higher than previously described for eoCRC; however, the *APC* mutations that were observed were distributed throughout the gene rather than at hotspots typically associated with sporadic CRCs (Aitchison et al., 2020[2]). 

Prior studies have also revealed some epigenetic differences in eoCRCs. *Long Interspersed Nuclear Element-1 *(*LINE-1*) hypomethylation is often used as a surrogate marker for global hypomethylation and is associated with CIN. In comparison to Lynch syndrome tumors, microsatellite stable (MSS) loCRCs or MSI-H loCRCs, eoCRCs showed significantly lower *LINE-1* methylation and patients demonstrated poor survival (Antelo et al., 2012[6]). Molecular pathways that drive the pathogenesis of CRCs are typically thought of in terms of CIN without MSI, or MSI without CIN, but a third group of CRCs denoted as microsatellite and chromosomally stable (MACS) have been observed at higher frequencies among eoCRCs (Banerjea et al., 2009[11]; Boardman et al., 2007[14]). These studies showing MACS utilized tissue samples collected prior to 1997 and might not fully capture molecular alterations driving the contemporary rise of eoCRC cases. It should be noted that with the elimination of Lynch syndrome and any somatic DNA mismatch repair defect through germline genetic analyses, there are no cases of eoCRC that follow the MSI-H pathogenic pathway.

Transcriptome alterations are also noted among eoCRCs. Holowatyj et al. investigated transcriptome profiling of MSS tumors and showed that comparing 34 MSS eoCRCs to 199 MSS loCRCs that eoCRCs contain an enrichment in the nuclear factor erythroid-2 related factor (*NRF2*) pathway, linking eoCRC pathogenesis to an oxidative stress response (Holowatyj et al., 2020[52]). Separately, immune profiling of 40 eoCRCs (compared to 39 loCRCs) revealed *SAA1*, *C7* and *CFD* as differentially expressed transcripts. With adjustment for clinicopathological features, higher expression of CFD and SAA1 were associated with worse progression-free survival; gain-of-function CFD expression in a mouse model enlarged tumor volumes and impacted multiple genes involved in immune regulation (Gardner et al., 2021[42]).

Overall, few papers have investigated the molecular signatures of sporadic eoCRCs, and the field is rapidly evolving. At present, there is no known distinct, unifying pathway that describes or defines eoCRC. There are no published studies to date that provide a direct link between the observed patient epidemiologic risk factors with any molecular changes among eoCRCs. It is possible that these investigations are still in their infancy and have yet to identify any unique molecular features in eoCRC. On the other hand, it may be that with the broad array of contributing risk factors, eoCRC may prove to be a biologically heterogenous disease without a single descriptive mechanistic pathway 

## Conclusion and Future Directions

There is a growing body of evidence of identifiable risk factors associated with eoCRC, with consistent findings of adiposity, diabetes, Western dietary patterns, sedentary lifestyle, smoking and moderate to heavy alcohol use as risk factors for eoCRC (Table 2(Tab. 2)). These risk factors are also associated with CRC in general. A handful of translational studies have investigated for unique altered transcriptomic, genomic and epigenomic differences among eoCRCs that might contribute to its pathogenesis. To date, there is no identifiable direct connection between observed eoCRC risk factors and any specific molecular mechanism. There is a particular knowledge gap between eoCRC patients and gut microbiome composition, as well as probable metabolic and inflammatory alterations that occur in eoCRC patients. The number of investigations for molecular alterations among eoCRC patients have been increasing, lending to the possibility of having meaningful relevant and actionable data on the horizon for eoCRC. 

There are minimal data available as to how risk factors can be utilized to risk stratify younger patients for intervention such as with targeted use of colonoscopy to prevent eoCRC. At present, widely accepted risk-based criteria to alter the age to initiate CRC screening include elements of the family history, and the presence of IBD or known genetic cancer syndromes. Only a minority (~20 %) of patients that develop CRC before the age of 50 years demonstrate any germline evidence of an inherited disease, and <1 % show any evidence of IBD. Some modifiable lifestyle-associated risk factors may impart a similar magnitude of risk as family history which prompts for early initiation of CRC screening. Further studies may establish how best to integrate a comprehensive risk assessment that allows for improved medical decision-making and the targeting of screening resources to maximize impact in minimizing eoCRC development. 

Colonoscopy saves lives through prevention and early detection of CRC. Patients who develop eoCRC show higher mortality and have more life-years lost but would likely have improved survival with early detection in pre-symptomatic stages of the disease. With the increase in eoCRC, average risk CRC screening will now encompass 45-49 year old patients, but this broad recommendation for screening in this younger-than-50 year old population may not be the most ideal approach considering potential resource constraints for mass screening in this new larger population pool to screen, versus a targeted or risk-stratification approach for the younger patients based on unique aspects of eoCRC that might be gained through the study of these patients and their tumors. 

## Declaration

### Disclosure of potential conflicts of interest

The authors declare that they have no conflict of interest.

### Acknowledgments

This work was supported by the United States Public Health Service (R01 CA258519 and T32 DK094775) and the Rogel Cancer Center of the University of Michigan. The funders had no role in study design, data collection and analysis, decision to publish, or preparation of the manuscript. 

## Figures and Tables

**Table 1:**
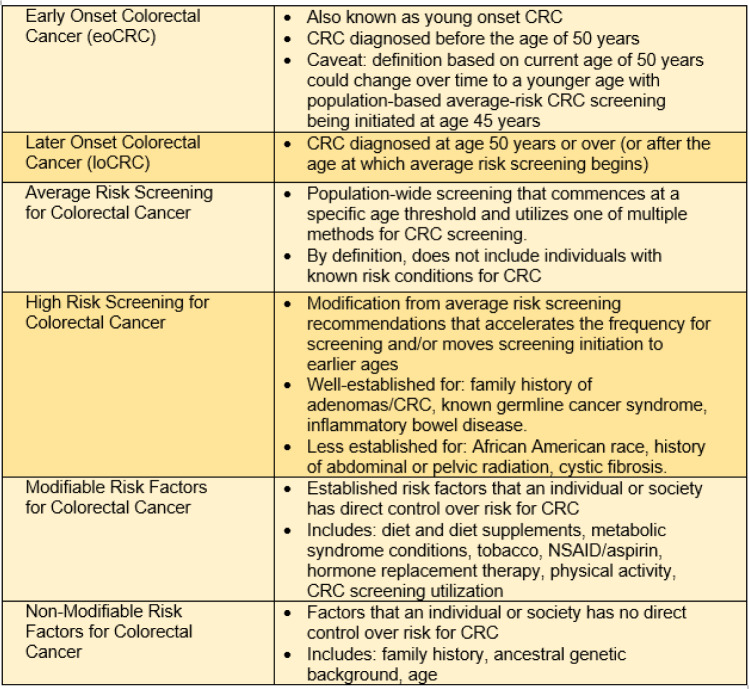
Definitions and key terms for early onset colorectal cancer

**Table 2:**
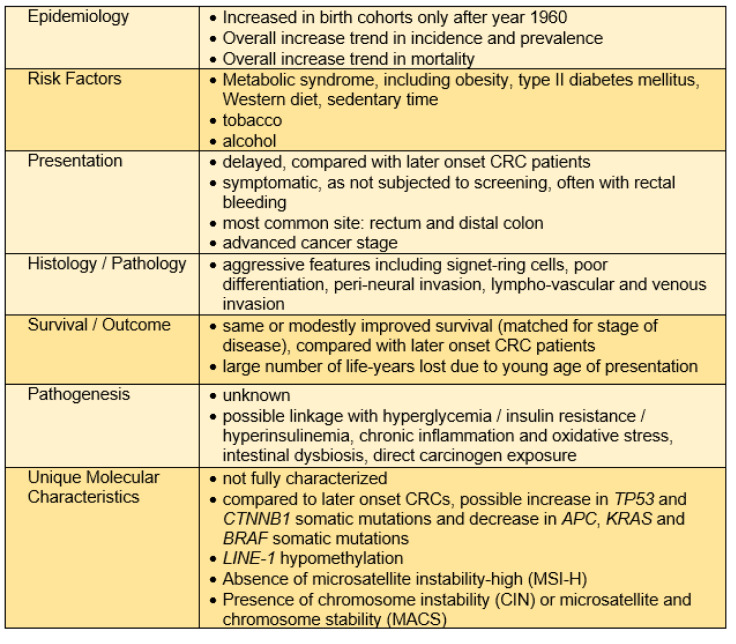
Key association factors with early onset colorectal cancer
